# Hao1 Is Not a Pathogenic Factor for Ectopic Ossifications but Functions to Regulate the TCA Cycle In Vivo

**DOI:** 10.3390/metabo12010082

**Published:** 2022-01-15

**Authors:** Atsushi Kimura, Akiyoshi Hirayama, Tatsuaki Matsumoto, Yuiko Sato, Tami Kobayashi, Satsuki Ikeda, Midori Maruyama, Mari Kaneko, Mayo Shigeta, Eri Ito, Tomoya Soma, Kana Miyamoto, Tomoyoshi Soga, Masaru Tomita, Akihito Oya, Morio Matsumoto, Masaya Nakamura, Arihiko Kanaji, Takeshi Miyamoto

**Affiliations:** 1 Department of Orthopedic Surgery, Keio University School of Medicine, 35 Shinano-machi, Shinjuku-ku, Tokyo 160-8582, Japan; kim_bierhoff@yahoo.co.jp (A.K.); roomkey0215@hotmail.co.jp (T.M.); bapybapy@hotmail.com (Y.S.); tami.koba@a8.keio.jp (T.K.); s00-022@nms.ac.jp (A.O.); morio@a5.keio.jp (M.M.); masa@keio.jp (M.N.); hikokanaji@gmail.com (A.K.); 2 Institute for Advanced Biosciences, Keio University, 246-2 Mizukami, Kakuganji, Tsuruoka 997-0052, Yamagata, Japan; hirayama@ttck.keio.ac.jp (A.H.); satsuki@ttck.keio.ac.jp (S.I.); la52la.green@gmail.com (M.M.); soga@sfc.keio.ac.jp (T.S.); mt@sfc.keio.ac.jp (M.T.); 3 Department of Advanced Therapy for Musculoskeletal Disorders II, Keio University School of Medicine, 35 Shinano-machi, Shinjuku-ku, Tokyo 160-8582, Japan; 4 Department of Musculoskeletal Reconstruction and Regeneration Surgery, Keio University School of Medicine, 35 Shinano-machi, Shinjuku-ku, Tokyo 160-8582, Japan; 5 Laboratory for Animal Resources and Genetic Engineering, RIKEN Center for Biosystems Dynamics Research, 2-2-3 Minatojima-minamimachi, Chuo-ku, Kobe 650-0047, Hyogo, Japan; mari.kaneko@riken.jp (M.K.); mayo.shigeta@riken.jp (M.S.); 6 Institute for Integrated Sports Medicine, Keio University School of Medicine, 35 Shinano-machi, Shinjuku-ku, Tokyo 160-8582, Japan; b055a004n@yahoo.co.jp; 7 Department of Dentistry and Oral Surgery, Division of Oral and Maxillofacial Surgery, Keio University School of Medicine, 35 Shinano-machi, Shinjuku-ku, Tokyo 160-8582, Japan; soma-t-oams@a8.keio.jp; 8 Department of Orthopedic Surgery, Kumamoto University, 1-1-1 Honjo, Chuo-ku, Kumamoto 860-8556, Japan; kana2001@galaxy.ocn.ne.jp

**Keywords:** ossification of the posterior longitudinal ligament, ectopic ossification, hydroxyacid oxidase 1, tricarboxylic acid cycle

## Abstract

Ossification of the posterior longitudinal ligament (OPLL), a disease characterized by the ectopic ossification of a spinal ligament, promotes neurological disorders associated with spinal canal stenosis. While blocking ectopic ossification is mandatory to prevent OPLL development and progression, the mechanisms underlying the condition remain unknown. Here we show that expression of hydroxyacid oxidase 1 (Hao1), a gene identified in a previous genome-wide association study (GWAS) as an OPLL-associated candidate gene, specifically and significantly decreased in fibroblasts during osteoblast differentiation. We then newly established Hao1-deficient mice by generating Hao1-flox mice and crossing them with CAG-Cre mice to yield global Hao1-knockout (CAG-Cre/Hao1flox/flox; Hao1 KO) animals. Hao1 KO mice were born normally and exhibited no obvious phenotypes, including growth retardation. Moreover, Hao1 KO mice did not exhibit ectopic ossification or calcification. However, urinary levels of some metabolites of the tricarboxylic acid (TCA) cycle were significantly lower in Hao1 KO compared to control mice based on comprehensive metabolomic analysis. Our data indicate that Hao1 loss does not promote ectopic ossification, but rather that Hao1 functions to regulate the TCA cycle in vivo.

## 1. Introduction

Ossification of the posterior longitudinal ligament (OPLL) is a progressive disease characterized by ectopic ossification in the PLL [1,2,3,4]. Patients with OPLL exhibit various neurological symptoms due to spinal canal stenosis occurring as ossification progresses [3,4]. OPLL is a rare disease and its underlying mechanisms are unclear, presenting a roadblock to devising means to inhibit ectopic ossification or its progression. OPLL patients who experience neuropathy or paralysis sometimes undergo surgery to enlarge the stenosed spinal canal. However, disease ossification activity can remain or recur after surgery, as enlarged spinal canals may also frequently re-stenose due to disease progression, requiring additional surgery to mitigate spinal canal closure [5,6,7,8]. Thus, the development of strategies to prevent OPLL by defining underlying disease mechanisms is mandatory.

Tiptoe walking (ttw) mice, which exhibit homozygous loss of ectonucleotide pyrophosphatase/phosphodiesterase 1 (Enpp1) function, spontaneously develop OPLL and are frequently utilized as OPLL models [9,10,11,12]

Comparable mutations in ENPP are also reportedly seen in human OPLL patients [9,13]. Ttw mice also exhibit hypophosphatemic rickets symptoms such as hypophosphatemia and low bone mass [14]. In humans, ENPP1 mutation causes autosomal recessive hypophosphatemic rickets (ARHR) or generalized arterial calcification of infancy (GACI), in which ectopic calcification occurs in various tissues (e.g., aorta) [15,16,17,18]. OPLL patients reportedly show lower serum phosphate levels than normal subjects [19,20,21]. Moreover, OPLL development is often seen in hypophosphatemic rickets patients as they age [22,23].

Ectopic ossification in ttw mice is reportedly caused by abnormally elevated vitamin D signaling, and phenotypes seen in ttw mice are completely abrogated when ttw mice are crossed with vitamin D receptor (VDR)-deficient mice [14].Ectopic ossification is also observed in patients with fibrodysplasia ossificans progressive (FOP) [24,25]. Activating mutations in the gene encoding activin receptor 2 (ACVR2) likely promote FOP development [26]. However, FOP patients reportedly do not show complications associated with OPLL.

Genome-wide association studies (GWASs) have been undertaken to identify candidate genes associated with OPLL development [2,9,27,28]. For example, one GWAS analysis, which included 1130 OPLL patients and 7135 controls, identified six loci—8p11.21, 8q23.1, 8q23.3, 12p12.2, 12p11.22 and 20p12.3—significantly associated with OPLL development [29]. Within these loci were genes encoding hydroxyacid oxidase 1 (HAO1), R-Spondin 2 (RSP2), EIF3E, CCDC91, EIF3H, CDC51, RSPHA and TMEM151B. Rsp2 regulates bone formation [30], and a single nucleotide polymorphism in *Rsp2* reportedly promotes enchondral ossification [31]. Nonetheless, how or whether these genes function in OPLL development or ectopic ossification is unknown.

Among these candidate factors, glycolate oxidase hydroxyacid oxidase 1 (encoded by Hao1) is expressed in the liver where it converts α-hydroxy acids to α-keto acids [32,33,34], concomitantly producing H_2_O_2_ [32]. Hao1 plays a role in oxalic metabolism [33,34], but Hao1-deficient mice have not been previously generated. Thus, Hao1 function in vivo remained untested.

In this study, we analyzed the expression of the above-mentioned eight candidate genes associated with OPLL development and found that only the level of Hao1 significantly changed and decreased during osteoblastic differentiation in fibroblasts. We then newly established Hao1-deficient mice and observed that, while Hao1 loss did not promote ectopic ossification or calcification in vivo, urinary levels of TCA cycle metabolites significantly decreased in Hao1-deficient mice compared with control mice, based on comprehensive metabolomic analysis.

## 2. Results

### 2.1. Hao1 Expression Significantly Decreased with Osteoblastic Differentiation

**Figure 1:**
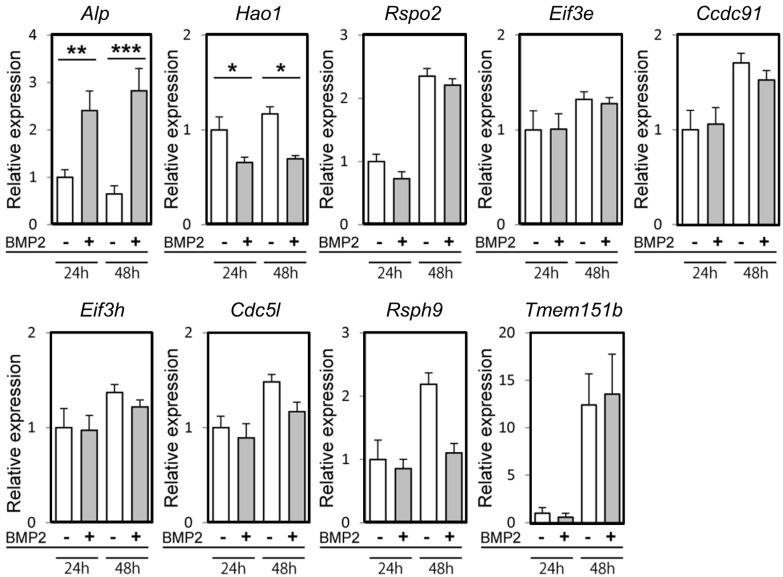
Hao1 expression was downregulated during osteoblastic differentiation of fibroblasts. Expression of *Alp* and eight candidate genes in mouse embryonic fibroblasts cultured in osteoblastic differentiation conditions (namely, in the presence or absence of 50 ng/mL of BMP2) for 24 or 48 h, as analyzed by real-time PCR. Data represent mean values of indicated parameters ± S.D. (* *p* < 0.05; ** *p* < 0.01; *** *p* < 0.001).

To analyze transcript levels of the eight candidate genes associated with OPLL development based on GWAS analysis, we evaluated the expression of each in osteoblastic cells in vitro (Figure 1). To do so, we cultured fibroblasts and subjected them to osteoblastic differentiation in the presence or absence of bone morphogenetic protein 2 (BMP2) for either 24 or 48 h. We then assessed the expression of each gene by real-time PCR (Figure 1). Among the genes tested, only Hao1 showed a significant change in gene expression levels at both time points. Its expression significantly decreased in differentiated cells (Figure 1), in marked contrast with that of the positive control alkaline phosphatase (*Alp*), a marker of osteoblast differentiation (Figure 1).

### 2.2. Generation of Hao1 Knockout Mice

**Figure 2:**
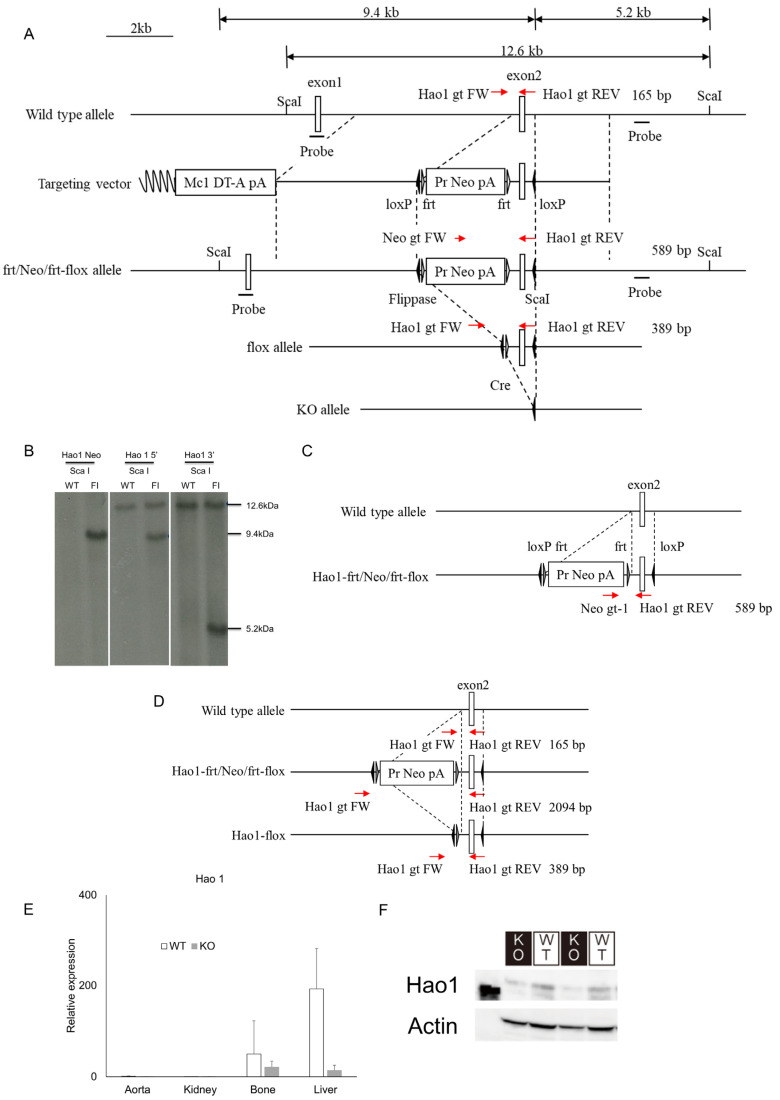
Establishment and characterization of Hao1 conditional knockout models. (**A**–**D**) We generated a targeting vector in which Hao1 exon 2 was flanked by loxP sequences and used it to transduce ES cells by homologous recombination (**A**). Neomycin-resistant colonies were picked and homologous recombination at the target site confirmed by Southern blot analysis of Sca1-digested genomic DNA. Blots were hybridized to either a 5′ or 3′ probe, identifying respective 9.4 kb or 5.2 kb fragments (**B**). The neomycin-resistant cassette flanked by frt sequences was deleted using flippase enzyme (**C**,**D**), and resultant ES cells were implanted into surrogate mothers. Hao1-flox F1 chimeric mice were obtained and bred to generate homozygous Hao1-flox mice. (**E**) Aorta, kidney, bone and liver were collected from ten-week-old WT and Hao1-deleted CAG-Cre/Hao1^flox/flox^ (KO) mice, and Hao1 mRNA expression was analyzed by real-time PCR. Shown is relative Hao1 expression in indicated tissues compared with that in wild-type aorta ± S.D. (**F**) Liver was collected from ten-week-old WT and Hao1-deleted CAG-Cre/Hao1flox/flox mice and Hao1 protein expression was analyzed by Western blotting. Red arrow symbols means the direction of each primer.

Since Hao1 inhibition accompanies osteoblastic differentiation, we established Hao1-deficient mice to assess whether decreased Hao1 expression promotes osteoblastic differentiation in fibroblastic tissues (e.g., ligaments) in vivo (Figure 2). To do so, we generated a targeting vector in which Hao1 exon 2 was flanked by loxP sequences, and used it to transduce ES cells by homologous recombination (Figure 2A). Neomycin-resistant colonies were picked and homologous recombination at the target site was confirmed by Southern blot analysis of Sca1-digested genomic DNA and hybridization to either a 5′ or 3′ probe, identifying respective 9.4 kb or 5.2 kb fragments (Figure 2B). The neomycin-resistant cassette flanked by frt sequences was deleted using flippase-expressing mice (Figure 2C,D), and resulting heterozygous Hao1-flox mice were bred to generate homozygous Hao1-flox mice. Those mice were crossed with CAG-Cre mice, in which Cre recombinase is induced under control of the chicken actin promoter globally, to yield global Hao1-deleted CAG-Cre/Hao1flox/flox mice (hereafter called Hao1 KO). Hao1 expression was analyzed in the aorta, kidney, bone or liver tissue of ten-week-old Hao1 KO and wild-type mice (WT), and judged to be successfully ablated in Hao1 KO mice based on real-time PCR (Figure 2E). Deletion of the Hao1 protein in the liver of Hao1 KO mice was confirmed by Western blot (Figure 2F).

### 2.3. Hao1 KO Mice Did Not Show Ectopic Ossification or Calcification

**Figure 3:**
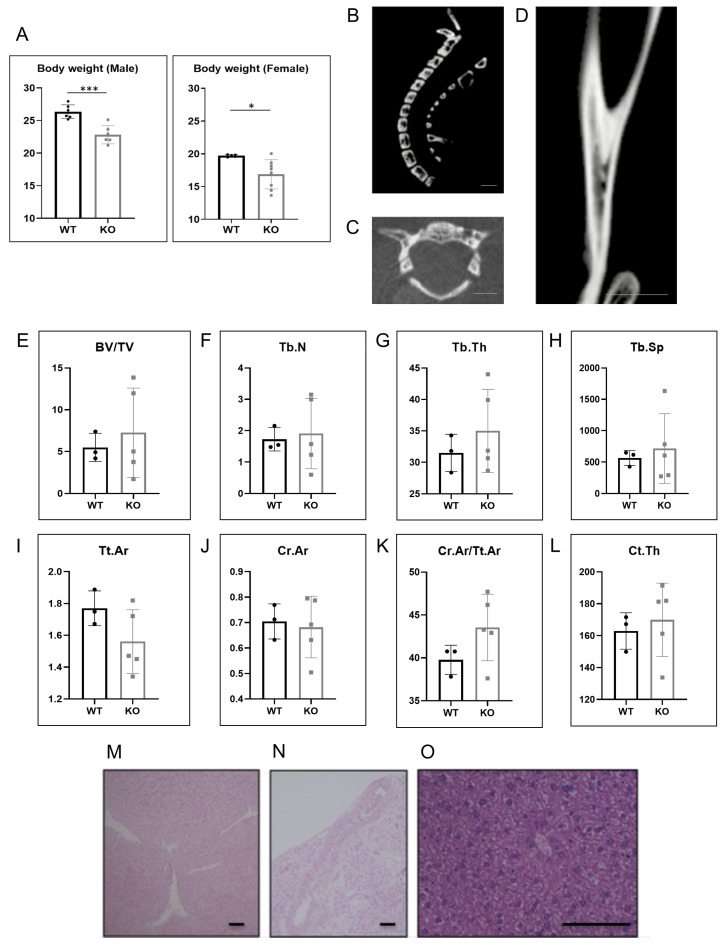
Body weight, micro CT, bone morphology, and histological evaluation. Eight-week-old Hao1-deleted CAG-Cre/Hao1^flox/flox^ (KO) mice were fed a high-phosphate diet for 2 weeks, and sacrificed at 10 weeks of age. (**A**) Comparison of body weights of male and female WT and KO mice at 10 weeks of age. Data represent mean values of indicated parameters ± S.D. (* *p* < 0.05; *** *p* < 0.001). (**B**,**C**) Micro CT analysis of sagittal (**B**) and axial (**C**) slices of spinal column. Scale bar = 2 mm. (**D**) Micro CT analysis of sagittal slice of tibia. Scale bar = 2 mm. (**E**–**L**) Bone morphology analysis of distal femoral metaphysis. (**M**–**O**) Von Kossa-stained images of kidney (**M**) and aorta (**N**) and of HE-stained liver (**O**). Scale bars = 100 µm.

Hao1 KO mice were fertile and exhibited no obvious abnormalities such as significant body weight loss (Figure 3A). Micro CT analysis revealed no evidence of ectopic bone formation, such as that seen in OPLL patients, in ten-week-old Hao1 KO mice (Figure 3B,C). Significantly, while ttw mice exhibit ectopic ossification in the Achilles tendon—a condition exacerbated by feeding a high-phosphate diet [9]—we did not observe comparable phenotypes in ten-week-old Hao1 KO mice, even after feeding mice a high-phosphate diet for two weeks (Figure 3D). Ttw mice also demonstrate relatively low bone mass [14]; however, Hao1 KO mice showed normal bone mass based on micro CT analysis of BV/TV, Tb.N, Tb.Th, Tb.Sp, Tt.Ar, Ct.Ar, Ct.Ar/Tt.Ar and Ct.Th (Figure 3E–L). Furthermore, although ttw mice show ectopic calcification in kidney and aorta [14], Von Kossa staining analysis did not reveal comparable phenotypes in Hao1 KO mice (Figure 3M,N). Additionally, although Hao1 is highly expressed in normal mouse liver (Figure 2E), we observed no abnormalities in the livers of Hao1 KO mice, based on HE staining (Figure 3O).

### 2.4. Hao1 Functions in Regulation of the TCA Cycle

**Figure 4:**
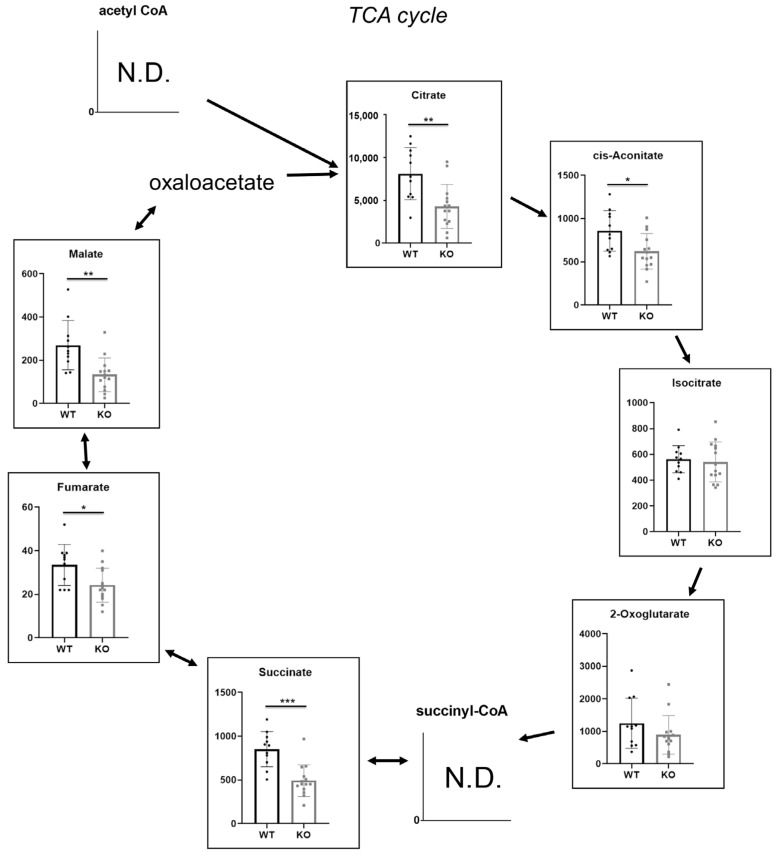
Metabolome analysis of urine from ten-week-old Hao1 KO and wild-type mice. Data represent mean values of indicated parameters ± S.D. (* *p* < 0.05; ** *p* < 0.01; *** *p* < 0.001).

Finally, we undertook metabolomic analysis to determine whether Hao1 enzymatically regulates metabolic pathways in vivo. To do so, we obtained urine samples from Hao1 KO and control mice at ten weeks of age and then compared indicated TCA metabolites (Figure 4) using a comprehensive metabolome analysis. Interestingly, we found that most TCA cycle metabolites were significantly lower in Hao1 KO mice than in controls (Figure 4).

## 3. Discussion

Patients with OPLL exhibit ectopic ossification, although the mechanisms underlying why this process occurs in fibroblastic soft tissue have remained unclear. In the spine, OPLL is most frequently seen in the highly mobile cervical spine, so mechanical stresses have been proposed as explanations for OPLL development [35,36]. However, ectopic ossification is not seen in relatively mobile limb ligaments, suggesting that other factors promote ectopic ossification in ligaments. Analysis of ttw mice suggests that systemic failure in phosphate metabolism and vitamin D homeostasis underlies ectopic ossification and calcification [9,15,37]. Indeed, serum phosphate levels are reportedly lower in OPLL patients than in normal subjects [19,20], and OPLL development is seen with aging in patients with hypophosphatemic rickets [21,22,23]. The results of this study suggest that Hao1 expression is significantly inhibited as fibroblasts undergo osteoblastic differentiation in vitro, but that Hao1 loss in vivo does not promote ectopic ossification and calcification. Instead, we observed that Hao1 is required to regulate the TCA cycle in vivo.

OPLL is an inherited disease [38]. Thus, we evaluated genetic factors for potential involvement in its development. GWAS has been used to identify unknown genetic factors underlying the development of diseases such as adolescent idiopathic scoliosis and rheumatoid arthritis [39,40,41,42]. In OPLL, R-Spondin2 was identified by GWAS and shown to stimulate endochondral ossification [29,30], but its role in inducing ectopic ossification in vivo was not clear. We show that Hao1 is predominantly expressed in liver (Figure 2E). *Hao2* is also reportedly expressed in the liver. Both Hao1 and Hao2 oxidize 2-hydroxy fatty acids [43] and thus may compensate for each other, which could underlie, at least in part, the lack of OPLL phenotypes seen in Hao1 KO mice. Indeed, Hao1*^−/−^* mice were established by others by deleting exon 3 of the Hao1 gene to yield Hao1 global knockout, and reportedly developed normally without significant phenotypes [44]. Nonetheless, to date, a function for fatty acids in OPLL development has not been demonstrated, and further studies are needed to define the function of Hao1 in OPLL development.

At sites of ectopic ossification, the trans-differentiation of soft tissue cells into osteoblasts has been reported [45]. Klotho mice are models of premature aging and exhibit aging-related phenotypes such as osteoporosis and short lifespan [46,47]. They also exhibit Monckeberg sclerosis as well as ectopic calcification and trans-differentiation of soft tissue cells into osteoblasts in the tunica media of the aorta in that ectopically calcified region [48]. Ttw mice fed a high-phosphate diet exhibited phenotypes almost identical to those seen in Klotho mice, which also develop OPLL [14]. These findings support the idea that the trans-differentiation of fibroblastic ligament cells into osteoblasts may underlie OPLL development.

Here, we present evidence in vivo that Hao1 functions to regulate the TCA cycle [49], based on significantly lower levels relative to controls of metabolites produced at various steps. At present, how Hao1 regulates the TCA cycle is not known. Additionally, to our knowledge, it has not previously been shown that perturbing the TCA cycle promotes ectopic ossification. Nonetheless, clarification of the mechanisms underlying ectopic ossification could identify novel therapeutic targets useful to treat or prevent OPLL.

## 4. Materials and Methods

### 4.1. Mice

C57BL/6 mice were purchased from Sankyo Labo Service (Tokyo, Japan). Mice were placed under specific-pathogen-free conditions in animal facilities certified by the Keio University Institutional Animal Care and Use Committee and the Institutional Animal Care and Use Committee of RIKEN Kobe Branch; they were maintained in an environment in accordance with Institutional Guidelines on Animal Experimentation at Keio University and with RIKEN Regulations for Animal Experiments. All animal experimental protocols were approved by the aforementioned committee and performed in accordance with their guidelines.

### 4.2. Quantitative Real-Time PCR

Mouse embryonic fibroblasts were cultured in the presence or absence of 50 ng/mL bone morphogenetic protein 2 (BMP2; Pepro Tech Ltd., East Windsor, NJ, USA). After 24 or 48 h of cultivation, total RNA was isolated and first-strand cDNA was generated using reverse transcriptase (Wako Pure Chemicals Industries, Osaka, Japan). Quantitaive RT-PCR was then performed using SYBR Premix ExTaq II reagent and a DICE thermal Cycler Real Time System III (Takara Bio Inc., Shiga, Japan). *β-Actin* (*Actb*) expression was analyzed as internal control. The following primers were used.

*Actb*—forward: 5′-TGAGAGGGAAATCGTGCGTGAC-3′
*Actb*—reverse: 5′-AAGAAGGAAGGCTGGAAAAGAG-3′
*Alp*—forward: 5′-CACCATTTTTAGTACTGGCCATCG-3′
*Alp*—reverse: 5′-GCTACATTGGTGTTGAGCTTTTGG-3′
Hao1—forward: 5′-CTTGCTGAATATGTGGCACAAGC-3′
Hao1—reverse: 5′-TAACAGCTTCCTTGGCATCATCA-3′
*Rspo2*—forward: 5′-AGCCAGCAAAAGACACAATACCAT-3′
*Rspo2*—reverse: 5′-TTCTCTTTTGCCTTTGGTGTTCTCA-3′
*Eif3e*—forward: 5′-CAGAACCAATCGTGAAGATGTTTG-3′
*Eif3e*—reverse: 5′-TCTTGCCTAAACCCATGTTTGTCT-3′
*Ccdc91*—forward: 5′-CCATCGAGAAGCAATATGTGTCTG-3′
*Ccdc91*—reverse: 5′-TGAGTCAAAGCTTCCTGGATTTTC-3′
*Eif3h*—forward: 5′-GGATAAGCACGAATTGCTCAGTCT-3′
*Eif3h*—reverse: 5′-GCGCATGTACGTGTTGTATTTGAT-3′
*Cdc5l*—forward: 5′-TGTGGGAGGAATGCTACAGTCA-3′
*Cdc5l*—reverse: 5′-TCTGTTGTCATGTGACCCCTGT-3′
*Rsph9*—forward: 5′-GTTGCAGAAGGTTAACGAAGGAGA-3′
*Rsph9*—reverse: 5′-AGCTACAGCCTTGTCAATCTGGTC-3′
*Tmem151b*—forward: 5′-CTGGTGGAAGGCCATCAGTTATC-3′
*Tmem151b*—reverse: 5′-TAGTCATCCAAGCCCTCGTTCTC-3′

### 4.3. Generation of Hao1 Conditional Knockout Mice

Hao1 conditional KO mice (Accession No. CDB1312K: http://www2.clst.riken.jp/arg/mutant%20mice%20list.html) were generated using C57BL/6-derived HK3i ES cells, as previously described [50]. To construct the targeting vector, genomic fragments of the Hao1 locus were obtained from a BAC clone (BACPAC Resources). A 722 bp region containing *Hao1* exon 2 was flanked by loxP sites (Figure 2). Targeted ES clones were microinjected into 8-cell-stage ICR embryos, and injected embryos were then transferred into pseudopregnant ICR females. Resulting chimeric mice were crossed with flippase-expressing mice (B6;SJL-TG(ACTFKPE)9205Dym/J), and heterozygous offspring were identified by PCR. Primers used were Hao1 gt FW (5′- GGATCAGCCCGATGGTACAAG-3′) and Hao1 gt REV (5′- CCTCTGACAAACATTGGTCTGC-3′) for the wild-type allele, and Neo-gt-1 (5′- CTGACCGCTTCCTCGTGCTTTACG-3′) and Hao1 gt REV for the Hao1-frt/Neo/frt-flox allele, and Hao1 gt FW and Hao1 gt REV for the Hao1-flox allele, yielding 165 bp, 589 bp and 389 bp products, respectively. The Hao1-flox mice were crossed with CAG-Cre mice [51] to yield global Hao1-deleted (CAG-Cre; Hao1^flox/flox^) mice. Cre-negative Hao1*^flox/flox^* littermates were analyzed as wild-type mice. The number of mice analyzed was previously described [52,53,54].

### 4.4. Metabolome Analysis

Frozen samples of urine from ten-week-old Hao1 KO and wild-type mice were thawed and 40 µL aliquots were mixed with 140 µL of Milli-Q water and 20 µL of an internal standard aqueous solution (containing methionine sulfone, trimesic acid, 3-aminopyrrolidine, 2 mM; PIPES, 4 mM; D-camphor-10-sulfonic acid, 200 µM). The solution was transferred onto a 5 kDa-cutoff filter (Human Metabolome Technologies, Tsuruoka, Japan) and filtered at 9100× *g* for 2 h at 4 °C. Filtrates were analyzed by capillary electrophoresis-time-of-flight mass spectrometry (CE-TOFMS).

CE-TOFMS analysis of cationic and anionic metabolites was performed as described in [55,56,57]. Briefly, cationic metabolites were separated on a fused-silica capillary (50 μm i.d. × 100 cm total length) filled with 1 M formic acid as the electrolyte, and methanol/water (50%, *v*/*v*) containing 0.01 μM hexakis (2,2-difluoroethoxy)phosphazene; Hexakis) was delivered as sheath liquid at a rate of 10 μL/min. Capillary temperature was maintained at 20 °C. The sample solution was injected at 5 kPa for 5 s, and a positive voltage of 30 kV was applied. ESI-TOFMS was conducted in the positive-ion mode, and the capillary, fragmentor, skimmer and Oct RF voltages were set at 4000, 75, 50 and 400 V, respectively. Nebulizer gas pressure was configured at 7 psig and the heated nitrogen gas (300 °C) supplied at a rate of 10 L/min. Anionic metabolites were separated using a commercially available COSMO (+) capillary (50 μm i.d. × 105 cm, Nacalai Tesque, Kyoto, Japan) filled with 50 mM ammonium acetate (pH 8.5) as electrolyte, and ammonium acetate (5 mM) in 50% (*v*/*v*) methanol/water containing 0.01 μM Hexakis was delivered as sheath liquid at a rate of 10 μL/min. The sample solution was injected at 5 kPa for 30 s, and a negative 30 kV was applied. ESI-TOFMS was conducted in the negative-ion mode, and the capillary, fragmentor, skimmer and Oct RF voltages were set at 3500, 100, 50 and 500 V, respectively. Other conditions were identical for cationic metabolite analysis. Mass spectra were acquired at a rate of 1.5 cycles/sec over a 50–1000 *m*/*z* range.

### 4.5. Analysis of Skeletal Morphology

Mice were sacrificed, and their hind limbs were removed and fixed in 70% ethanol. Changes in femoral bone microstructure were assessed using a micro-computed tomography (micro CT) system (CosmoScan GX; Rigaku corporation, Tokyo, Japan). The distal femur and diaphyseal femur were scanned at 90 kV, 160 mA, with a voxel size of 20 × 20 × 20 mm. Outcomes were assessed in cortical bone of the diaphyseal femur, based on methods described in a previous study [58]. The metaphysis was measured starting 200 mm below the growth plate and extending distally for 4000 mm, and the diaphyseal was measured over an area 500 mm wide.

### 4.6. Statistical Analysis

Statistical analysis was performed using an unpaired two-tailed Student’s *t*-test between groups (* *p* < 0.05; ** *p* < 0.01; NS, not significant, throughout the paper).

## Abbreviations

OPLL: ossification of the posterior longitudinal ligament; Hao1, hydroxyacid oxidase 1; Hao1 KO, Hao1-knockout (CAG-Cre/Hao1flox/flox, Hao1 KO) mice; Enpp1, ectonucleotide pyrophosphatase/phosphodiesterase 1; ttw, tiptoe walking; ARHR, autosomal recessive hypophosphatemic rickets; GACI, generalized arterial calcification of infancy; VDR, vitamin D receptor; FOP, fibrodysplasia ossificans progressive; ACVR2, activin receptor 2; GWAS, genome-wide association study.
